# Outbreaks of Ebola virus disease in Africa: the beginnings of a tragic saga

**DOI:** 10.1186/1678-9199-20-44

**Published:** 2014-10-03

**Authors:** Jean-Philippe Chippaux

**Affiliations:** UMR 216, Mother and Child Facing Tropical Diseases, Institut de Recherche pour le Développement (IRD), Cotonou, Bénin; Sorbonne Paris Cité, Faculté de Pharmacie, Université Paris Descartes, Paris, France

**Keywords:** Ebola, Outbreak, Virus, Hemorrhagic fever, Africa

## Abstract

The tremendous outbreak of Ebola virus disease occurring in West Africa since the end of 2013 surprises by its remoteness from previous epidemics and dramatic extent. This review aims to describe the 27 manifestations of Ebola virus that arose after its discovery in 1976. It provides an update on research on the ecology of Ebola viruses, modes of contamination and human transmission of the disease that are mainly linked to close contact with an infected animal or a patient suffering from the disease. The recommendations to contain the epidemic and challenges to achieve it are reminded.

**Electronic supplementary material:**

The online version of this article (doi:10.1186/1678-9199-20-44) contains supplementary material, which is available to authorized users.

## Introduction

The outbreak of Ebola virus disease (EVD) occurring in West Africa since December 2013 will mark the history – however brief – of the viruses. Occurring for the first time outside its original home – the Central African rainforest – this epidemic of EVD appears as the deadliest and longest of all those known so far. At this time (August 15th, 2014), more than 1,250 deaths have been reported, *i.e.* five times more than during the worst previous outbreak. In addition, its rapid propagation has led the World Health Organization (WHO) to declare on August 8th that EVD represent a “Public Health Emergency of International Concern” and urged the international community to take action to stop the spread. Finally, for the first time, in response to the severity of the situation, WHO agreed the use of experimental treatment against the EVD.

## Review

Discovered in 1976 during two inaugural epidemics, both in Sudan and Democratic Republic of Congo (DRC), the Ebola viruses were responsible for 27 occurrences in Africa before the current outbreak of Guinea. This review aims to remind the characteristics of the different epidemics of EVD that were reported between 1976 and 2013.

### Ebola viruses

The Ebola virus is, together with *Marburg marburgvirus*, native to East Africa, and belongs to the *Filoviridae* family (Table 1), whose newest member is *Lloviu cuevavirus*, recently isolated in Spain from bats[1–3].

**Table 1:** ***Filoviridae*** **virus occurrences in the world**

Viral species	Year of discovery	Geographic origin	Number of outbreaks	Number of human cases	Number of deaths (CFR)	CFR (%)
*Marburg marburgvirus*	1967	Uganda	4	465	145	31
*Sudan ebolavirus*	1976	Sudan	6	792	426	54
*Zaire ebolavirus*	1976	DR Congo	12	1,388*	1,100*	79
*Reston ebolavirus*	1989	Philippines	0	0	0	–
*Taï Forest ebolavirus*	1994	Côte d’Ivoire	0	1	0	–
*Bundibugyo ebolavirus*	2007	Uganda	2	208	78	38
*Lloviu cuevavirus*	2010	Spain	0	0	0	–

CFR: case fatality rate. *Excluding the current West African outbreak.

**Figure 1:**
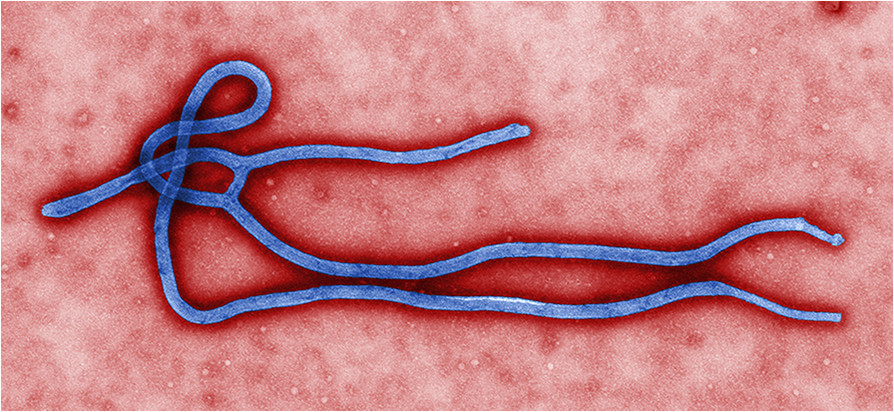
**Ultrastructural morphology of Ebola virus virion (image by US Centers for Disease Control and Prevention and Cynthia Goldsmith).**

Ebola viruses are RNA viruses whose genome encodes seven proteins[4, 5]. They are of filamentary form, sometimes branched, with a diameter of 80 nm and a length of up to 14,000 nm (Figure 1). The protein shell encloses the tubular helical nucleocapsid. Surface transmembrane glycoproteins of the virion provide the binding and fusion with the cell membrane, and penetration into the cell. Glycoproteins are responsible for almost all the virulence, even though it does not explain all the pathogenicity[6]. Monocytes, particularly macrophages, are the first cells infected, triggering apoptosis in lymphocytes[7]. Within three days, the virions invade the endothelial system[4, 5]. The inhibition of the immune response, including reduced production of interferon, favors the rapid spread of the virus in the body[8, 9].

The genus Ebola comprises five species, including the four African species involved in human clinical cases[3, 10]. Three of them were identified during Central African epidemics: first, *Sudan ebolavirus* and *Zaire ebolavirus*, both in 1976 respectively in Sudan and DRC, and more recently *Bundibugyo ebolavirus*, in 2007 in Uganda[11–14]. A fourth species, *Taï Forest ebolavirus*, was isolated from a primatologist who autopsied a chimpanzee in Côte d’Ivoire and survived the disease[15]. The last species, *Reston ebolavirus*, was isolated from monkeys native to Philippines farms[16]. The disease is deadly to monkeys. However, no human cases have been reported to date despite the presence of antibodies proving the infection in pigs (also without clinical illness) and humans[17].

**Figure 2:**
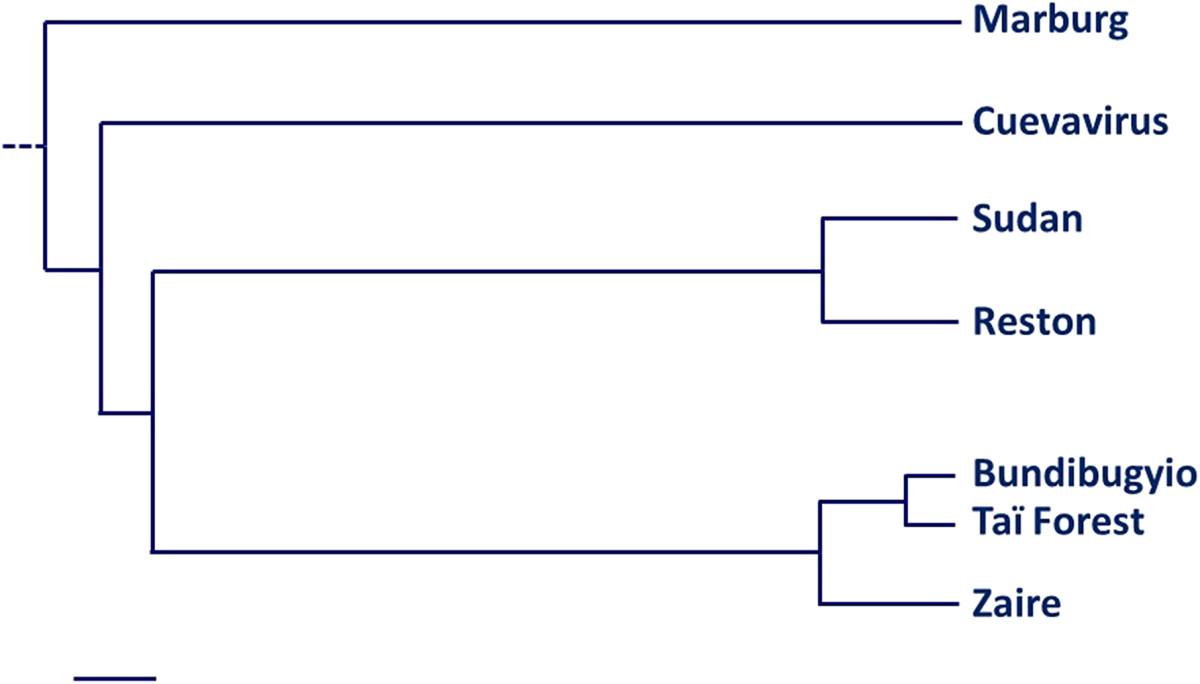
**Tentative of** ***Filoviridae*** **phylogeny (scale on bottom left represents about 100 years)**[2, 10, 17–20]**.**

According to Carroll *et al*.[10], the nearest common ancestor of Marburg and Ebola viruses dates back to 1,300 years. The separation of *Sudan ebolavirus* and other species of Ebola would have occurred soon after (about 1,200 years). The other species would have been separated much later, probably in the last hundred years or less (Figure 2).

### Clinical presentation

Incubation ranges from 3 to 21 days[21]. Eichner *et al*.[22] determined that the incubation period was 12.7 ± 4.3 days, which fixes the average time between two generations of patients. The epidemic is considered complete after an interval of at least twice the maximum incubation period, *i.e.* 42 days after the death or recovery of the last confirmed case[23].

The disease lasts 5 to 15 days[24]. It begins suddenly with fever, headache, abdominal pain, arthralgia and myalgia, therefore the general picture resembles a flu syndrome[25–29]. About half of patients complain of cough and sore throat with dysphagia. Digestive disorders (diarrhea, nausea, vomiting) follow in a variable proportion of patients. Hemorrhages occur in 30-80% of patients, mostly at the end of the illness, expressed by purpura, epistaxis, gingival bleeding, gastrointestinal bleeding or other, and appear to be associated with the severity of infection[24–30]. The variation in prevalence of symptoms, especially bleeding, is related to the viral species or clinical description that is based, according to the studies, on either suspected or confirmed cases.

Mortality is always high, although pathogenicity varies from one species to another (Table 1). In addition, some authors noted that mortality decreased during the epidemic. Several hypotheses have been advanced: loss of the virus virulence after successive generations, route of inoculation, viral load, improved management of cases and better enforcement of prophylaxis during the epidemic[24, 26, 30–33]. One cannot exclude that improving diagnosis in late epidemic reveals less severe cases.

### Diagnosis

**Figure 3:**
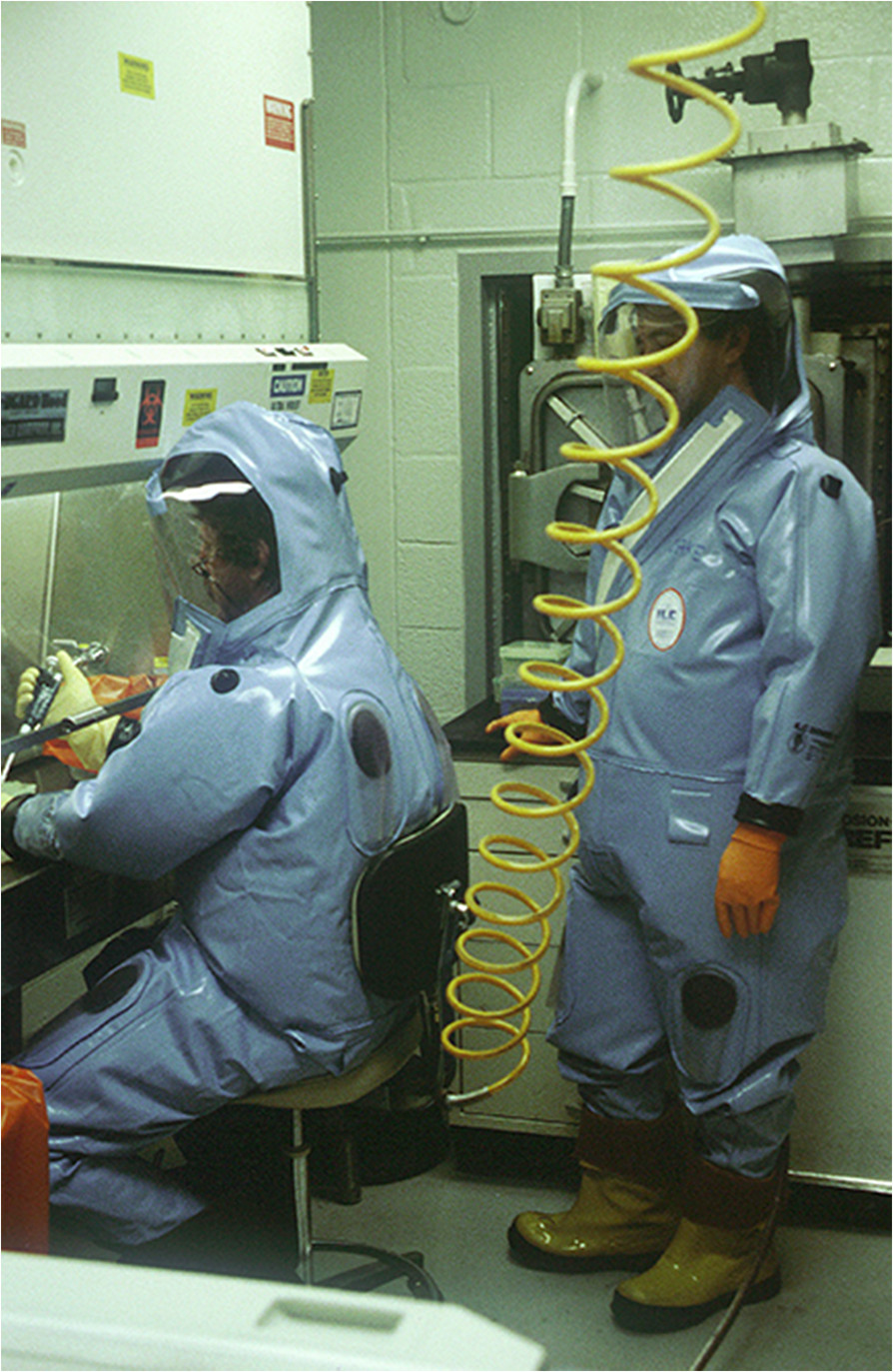
**Study of Ebola virus in a high-security laboratory BSL-4 (photo by IRD, ©IRD).**

The clinical diagnosis is difficult at the beginning of the epidemics, because of the poor specificity of the symptoms[24, 27, 29, 30, 33]. When the virus responsible for the outbreak is identified, all suspected cases should be considered as high risk and meet the case definition and exposure risks (Tables 2 and3) for better management of the epidemic. There is no carrier state.Laboratory diagnosis can only be performed in a specialized laboratory. First, there is no commercial reagent and, secondly, the samples represent an extreme biohazard that must be handled under containment conditions of the highest level (biosafety level 4 – BSL-4; Figure 3).

**Table 2:** **Case definition of Ebola Virus Disease (EVD)** [23, 34]

Name	Definition
**Index case**	Very first case (probable or confirmed, see below) found to be the origin of the outbreak
**Alert case**	Any person with sudden onset of high fever or sudden death or bleeding or bloody diarrhea or blood in urine
**Suspect case** (person under investigation)	Any person, dead or alive, who present (or presented before the death):
	(i) fever (>38.5°C or 101.5 °F) with additional symptoms (severe headache, muscle pain, vomiting, diarrhea, abdominal pain, or unexplained hemorrhage) and (ii) epidemiologic risk factors within the past 21 days before the onset of symptoms (close contact with body fluids of a suspect or probable case of EVD, or direct handling of bush animals from disease-endemic areas)
**Probable case**	Person with symptoms compatible with EVD, as evaluated by a clinician, or a dead person with an epidemiological link with a confirmed case
**Contacts**	Person without suggestive symptom of the disease, but who has been in contact with a suspect or probable case of EVD (living in the same house, provided care during the illness, participated in the burial rites etc.). It should be important to assess the risk level (see Table 3).
	If laboratory samples are obtained at an appropriate time during the illness, the previous notification categories should be reclassified as “laboratory-confirmed” cases and “not a case”
**Confirmed case**	Case with positive laboratory response for either Ebola virus antigen or Ebola IgG antibody
**“Not a case”**	Person with no Ebola-specific detectable antibody or antigen

**Table 3:** **Definition and assessment of risk exposure** [23, 34–36]

Risk level	Definition
**High-risk exposure**	• Percutaneous injury, e.g. needlestick, or mucous membrane exposure to body fluids of an EVD patient
	• Direct care or exposure to body fluids of an EVD patient without appropriate personal protective equipment (PPE)
	• Laboratory worker processing body fluids of confirmed EVD patients without appropriate PPE or standard biosafety precautions
	• Participation in funeral rites that include direct contact with human remains in the geographic area where an outbreak is occurring without appropriate PPE
**Low-risk exposure**	• Household member or other casual contact^1^ with an EVD patient
	• Providing patient care or casual contact^1^ without high-risk exposure with EVD patients in health care facilities in EVD outbreak affected countries
**No known exposure**	Persons with no known exposure were present in an EVD outbreak affected country in the past 21 days with no low-risk or high-risk exposures
	^1^Casual contact is defined as (i) being within approximately 3 feet (1 meter) or within the room or care area for a prolonged period of time (e.g. healthcare personnel, household members) while not wearing recommended personal protective equipment; or (ii) having direct brief contact (e.g., shaking hands) with an EVD case while not wearing recommended personal protective equipment

In patients, the diagnosis is carried out by the detection of viral antigens through ELISA, identification of nucleic acid by PCR, specific antibody titer, or virus isolation. Specific IgM and IgG antibodies appear during the second week following the first clinical signs (about 15 to 20 days after the infection). IgM titers persist about two months whereas IgG titers remain several years after the end of the disease[37].

Virus isolation can be achieved by inoculation in mice, guinea pigs or non-human primates in whom the disease is very close to what is observed in humans. The virus grows on kidney cell lines from African green monkey *Cercopithecus aethiops*. The diagnosis is made by optical microscopy examination of cytopathic effects, visualization of the virions by electron microscopy, identification of specific proteins by ELISA, or RNA detection by PCR.

IgG titration allows retrospective diagnosis in convalescents or exposed persons, or epidemiological investigations even years after the epidemic.

### Treatment

Antibodies acquired during the disease persist in survivors for more than ten years after the recovery. There is still no evidence that primary infection is protective in humans, apart from neutralization tests *in vitro*[38]. Moreover, up to this moment, there is no vaccine or effective treatment.

Studies on preventive vaccines are in progress[39, 40]. Some of them showed very good efficacy and are produced under good manufacturing practices conditions. Phase I trials demonstrated that the drug is safe for humans[40]. However, vaccine development is hampered by limited commercial interests, even taking into account the risks of bioterrorism-related dispersion of such virus. The limited number of cases, despite the high mortality, adds to the complexity – and cost – of large-scale immunization of a scattered and often inaccessible population[41].

Post-exposure management should consider either passive immunotherapy, or administering drugs that block the action of the virus or its replication. Convalescent sera, thought to contain natural specific protective antibodies developed during the disease, have been used exceptionally, with some success[42, 43]. It is noteworthy that these patients had also received better symptomatic treatments than regular patients, and were not representative. However, despite this indisputable bias, several experimental studies have confirmed, particularly in non-human primates, the effectiveness of such approach[44]. The development of specific drugs is underway with some promising molecules, including monoclonal antibodies, which use is related to passive immunotherapy[45]. Recently, Warren *et al*.[46] showed that a new nucleoside analogue protects against infection with *Filoviridae* by inhibiting the viral polymerase in the *Macacus cynomolgus* model that, moreover, seems to tolerate well the treatment.

Symptomatic treatments are poorly documented because of the rarity of the disease (less than 2,500 known cases worldwide between 1976 and 2013) and the paucity of resources in endemic countries, which limits the choice of drugs[47]. Administration of antihemorrhagic drugs, substitution treatments, including transfusions, plasmapheresis or dialysis, and resuscitation are anecdotal and concern very few patients[47]. Although one of the main virus targets, interferon has not been an effective treatment, which confirms that the impairment of the immune system is deep[4, 5, 47, 48]. Most often, palliative treatments are limited to rehydration with sugar solutions, preferably orally to avoid injections, analgesic, antipyretic, antiemetic, anti-diarrheal and sedatives or antipsychotic drugs to ease agitated and anxious patients.

### Natural history of ebola viruses

The emergence, less than 50 years ago, of Ebola virus remains an enigma. It is possible that sporadic cases or limited outbreaks occurred in the past and were unreported due to lack of epidemiological surveillance or appropriate diagnosis. Scattered and minor manifestations of EVD suggest that Ebola viruses circulate in a pathogen complex showing no (or few) contacts with human populations. Destruction of forests and human impact on broader areas could explain the increased frequency and severity of outbreaks[49]. However, we cannot exclude a recent emergence of the virus that would spread rapidly in a susceptible population[50]. According to Polonsky *et al*.[51], increasing frequency of epidemics may result from the combination of: improvement of monitoring and diagnostic capacities, increase of contact among humans and the natural reservoirs of the virus, and growth of the viral load and prevalence of the virus in reservoirs.

Several epidemiological investigations in Central and East Africa have shown circulation of Ebola virus in the human population at a significant rate, but that does not always entail the emergence of an epidemic[24, 52, 53].

**Figure 4:**
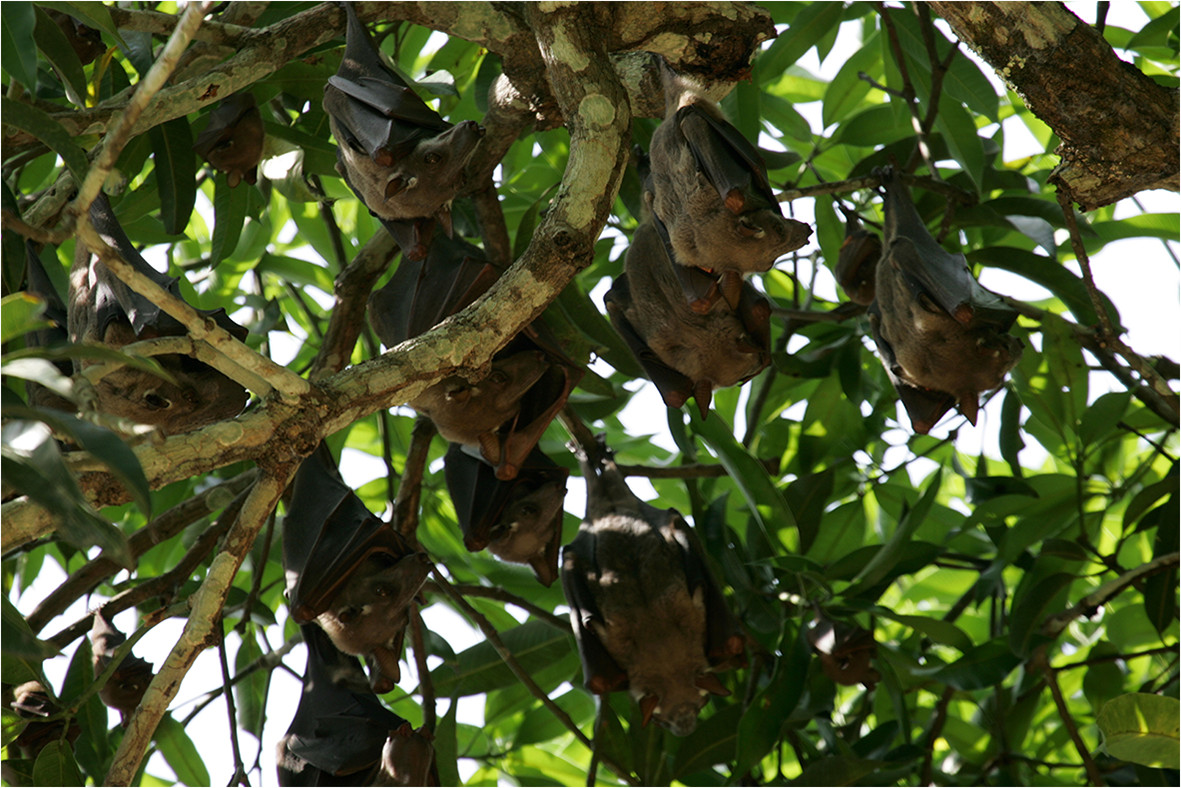
**Group of bats** ***Hypsignathus monstrosus*** **in a mango tree in Lambaréné, Gabon (photo by Jean-Jacques Lemasson, ©IRD).**

The natural reservoir of the virus is not known with certainty. Extensive investigations made in small mammals, even sensitive to the Ebola viruses, were negative during the various epidemics in Central Africa[26, 54–56]. Subsequent investigations continued outside epidemics. Although viral RNA and specific antibodies have been identified in small mammals, no potential natural host has been acknowledged until 2005[57]. However, initially dismissed due to many negative samples, fruit bats (Figure 4) were found with specific viral DNA and antibodies. These animals seem resistant to *Filoviridae* pathogenicity[18, 58–63].

The search for potential vectors, especially among arthropods, has always proved negative, including bedbugs (*Cimex hemipterus*) captured in the beds of infected persons[26, 54, 64, 65].

Deadly outbreaks of Ebola virus have been observed in non-human primates with high mortality[66]. In addition to the contamination of the Swiss primatologist in Ivory Coast[15], the index case of several outbreaks have been more or less directly associated with hunting or consumption of bush meat, *i.e.* monkeys, antelopes, bats[26, 58, 65, 67–71]. Natural infection of bats and sharing their narrow ecological niche with many species of non-human primates are strong arguments in favor of their role as a natural reservoir of the virus[58–61]. Some species, including *Eidolon helvum*, *Hypsignathus monstrosus*, *Myonycteris torquata* and *Epomops franqueti*, migrate long distances (>2,500 km), which could explain the multiple remote epidemic clusters[58, 72, 73].

Seasonal variation in mortality in chimpanzees of the Tai forest, Ivory Coast, and prevalence of specific antibodies against *Zaire ebolavirus* virus in febrile patients from East Africa suggests an influence of the climate in the occurrences of Ebola epidemics[66, 74]. Pinzon *et al*.[75] found a close relationship between the onset of epidemics and particularly dry conditions at the end of the rainy season, leading to a change in the behavior of fruit-eating mammals, particularly sensitive to weather changes, resulting in the increase of virus circulation or human contamination[76]. The seasons punctuate migration of bats, which could explain the emergence of epidemics[58, 62].

### Human transmission of Ebola viruses

The contamination of index cases is probably due to contact with an infected animal. Human transmission happens only through close contact with an ill or convalescent person, although at this stage the risk of infection is very small. Studies conducted during the various epidemics have shown that less than one fifth of the people (see Tables 2 and3) living with a confirmed or probable primary patient have developed the disease[24–26, 35]. All secondary cases were recorded among people with close contact with the patient and exposed to infected biological fluids. Conversely, people who had no contact with the patient were not sick. Such close contact with the patient throughout care occur mainly during the illness or burial preparation, including washing the body and funeral ritual that can be long and intimate[25, 26, 77–79]. The risk greatly increases due to the delay in diagnosis and appropriate management[24, 27, 29, 30, 33].

Ebola viruses have been detected in most patient secretions. They are present in the blood, saliva, feces, breast milk, tears and genital secretions. They have not been isolated from vomit, sputum, sweat or urine. However, the number of tested samples was low[80]. The virus persists in breast milk, genital secretions and glass during convalescence and up to 13 weeks after recovery[27, 80, 81]. Finally, the risk of transmission from fomites (towels, clothes and sheets from the patient), especially during convalescence, is low and basic protection measures are likely to be sufficient[80].

Nosocomial transmission is behind many hospital outbreaks[21, 24, 28]. Injection materials reused without precaution or inadequately sterilized have been repeatedly denounced and remain a major cause of epidemic spread. This also applies to traditional healers whose practices are often septic[67, 82].

Finally, all the studies performed during the outbreaks of EVD showed that contamination occurs due to close contact with the blood or secretions of an infected patient through three ways[25, 26, 35, 36]:

- Patient care – usually a family member takes care of the patient during the illness.
- Preparing the deceased for funeral before or during burial.
- Nosocomial transmission – reuse of medical equipment that has been previously used in a patient infected with Ebola virus.

There is no contamination by air or just handshake.

### Manifestations of Ebola viruses in Africa (1976–2012)

**Figure 5:**
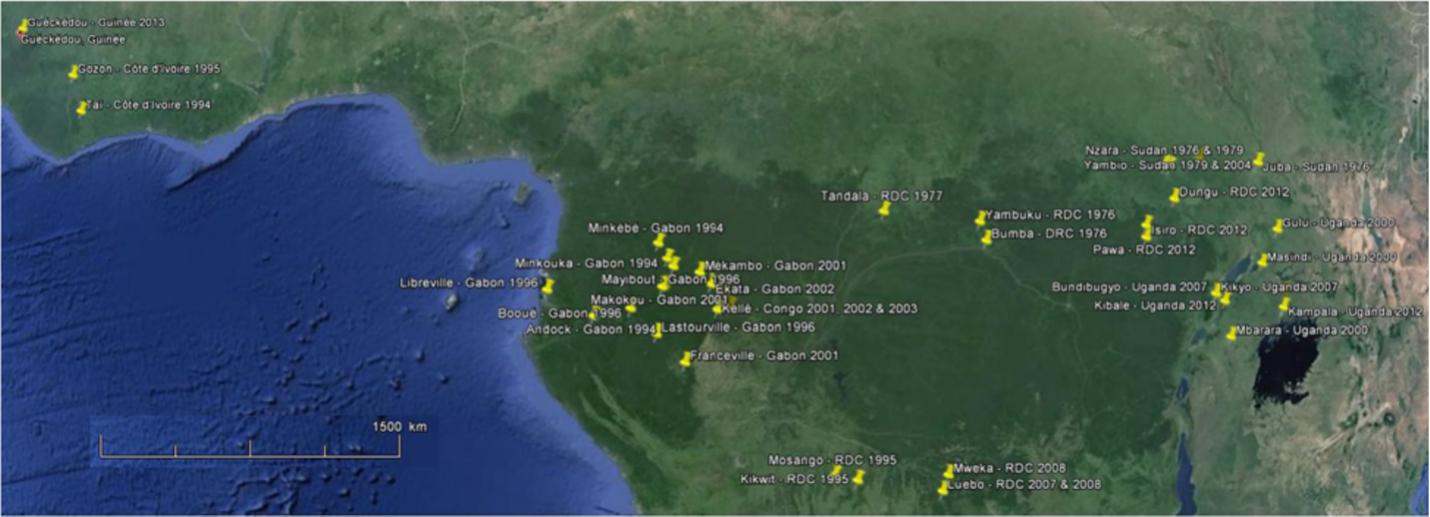
**Geographical distribution of African manifestations of Ebola viruses (based on Google™ Earth map).**

Since their discovery in 1976, simultaneously in Sudan and DRC, and until 2012, *i.e.* the recent outbreaks observed in Uganda and again in the DRC, Ebola viruses were notified 27 times (Table 4, Figure 5), including in 22 epidemics. It is possible that sporadic cases or limited outbreaks have escaped any mention due to the remoteness and poverty of the concerned people.

**Table 4:** **Characteristics of the African manifestations of Ebola virus (bolded names indicate the place of first case occurrence)**

Year	Country	Districts	Ebola species	Length (weeks)	Number of cases				Ways of transmission	References
					Presumed*	Confirmed	Deaths	Total		
1976	Sudan	**Nzara**, Maridi, Tembura, Juba	Sudan	22	227	57	151	284	Nursing patient	[12, 25]
1976	DRC	**Yambuku**, Abumombazi, Kinshasa	Zaïre	9	307	11	280	318	Nosocomial**	[11–13, 26]
									Nursing patient	
									Funeral/burial ritual	
1977	DRC	Tandala	Zaïre	–		1	1	1		[83]
1979	Sudan	**Nzara**, Yambio	Sudan	10	24	10	22	34	Nursing patient	[24]
1994	Gabon	**Minkouka**, Andock, Minkébé	Zaïre	13	32	19	31	51	Nursing patient	[67, 82, 84]
1994	Côte d’Ivoire	Taï	Taï Forest	–		1	0	1		[15]
1995	Côte d’Ivoire /Liberia	Gozon	?			1	0	1		[85, 86]
1995	DRC	**Kikwit**, Mosango (±30 villages)	Zaïre	27	233	82	255	315	Nosocomial**	[28, 87]
									Nursing patient	
									Funeral/burial ritual	
1996	Gabon	Mayibout	Zaïre	12	29	2	21	31	Eating bush meat	[67, 82, 88]
									Funeral/burial ritual	
1996	Gabon	**Booué**, Balimba, Lastourville, Libreville	Zaïre	27	56	4	45	60	Eating bush meat	[67, 82, 89–91]
									Nursing patient	
1996	South Africa	Johannesburg	Zaïre	–	0	2	2	2		[82]
2000	Uganda	**Gulu**, Masindi, Mbarara	Sudan	20	230	195	224	425	Nosocomial**	[21, 31]
									Nursing patient	
2001-2002	Gabon	**Mékambo** , Makokou, Franceville	Zaïre	21	37	28	53	65	Nosocomial**	[69, 82, 34]
									Nursing patient	
									Funeral/burial ritual	
2001-2002	Congo	Mbomo, Kellé	Zaïre	20?	50	9	44	59	Nosocomial**	[34]
									Nursing patient	
									Funeral/burial ritual	
2002	Congo	Mbomo	?	10	9		8	9	?	[34]
2002	Gabon	Ekata	?	10	2		2	2	?	[34]
2002-2003	Congo	Mbomo, Kellé	Zaïre	17	130	13	128	143	Nursing patient	[71]
									Funeral/burial ritual	
2003	Congo	**Mbomo**, Mbandza	Zaïre	7	18	17	29	35	Nursing patient	[92]
									Funeral/burial ritual	
2004	Sudan	Yambio	Sudan	10	4	13	7	17	Nursing patient	[93]
									Funeral/burial ritual	
2005	Congo	**Etoumbi**, Mbomo	Zaïre	6	11	1	10	12	Nursing patient	[70]
									Funeral/burial ritual	
2007	DRC	Luebo	Zaïre	17	≤ 170	≥ 17	186	264	No data	[47, 19]
2007	Uganda	**Bundibugy**, Kikyo	Bundibugyo	20	75	56	42	131	Nursing patient	[29, 30, 33, 94]
									Funeral/burial ritual	
2008	DRC	**Luebo**, Mweka	Zaïre	5	≤ 29	≥ 3	15	32	No data	[19]
2011	Uganda	Luwero	Sudan			1	1	1		[95]
2012	Uganda	Kibale	Sudan	11	13	11	17	24	No data	[96, 97]
2012	Uganda	**Luwero** , Kampala	Sudan	8	3	6	4	7	No data	[97–99]
2012	DRC	**Isiro**, Pawa, Dungu	Bundibugyo	29	41	36	36	77	Nosocomial**	[79, 100]
									Nursing patient	
									Funeral/burial ritual	

*Alert, suspected or probable case, *i.e.* diagnosis based on clinical and/or epidemiological criteria but not biological evidence (see Table 3). In some outbreaks, case definition changed during the epidemics.

**Hospital transmission due to needle and syringe contamination, contact with patient’s blood, secretions or fomites.

Some individual infections, such as those observed in Tandala (DRC) in 1977, Taï (Côte d’Ivoire) in 1995, Luwero (Uganda) in 2011, indicate an occult but permanent circulation of Ebola viruses[15, 83, 95]. The confirmed case in Gozon (Côte d’Ivoire) in 1995, which may have come from Liberia, has not been completely documented and is not mentioned after the year 1999[85, 86]. The index case in Johannesburg in 1996 was a patient infected in Gabon, where he was staying during the Ebola outbreak in that country[82]. Finally, some epidemics may be the expression of successive waves of the same epidemic, as in Gabon in 1996, Gabon and Congo between 2000 and 2003, and Uganda in 2012, confirming the persistence of the Ebola virus.

**Figure 6:**
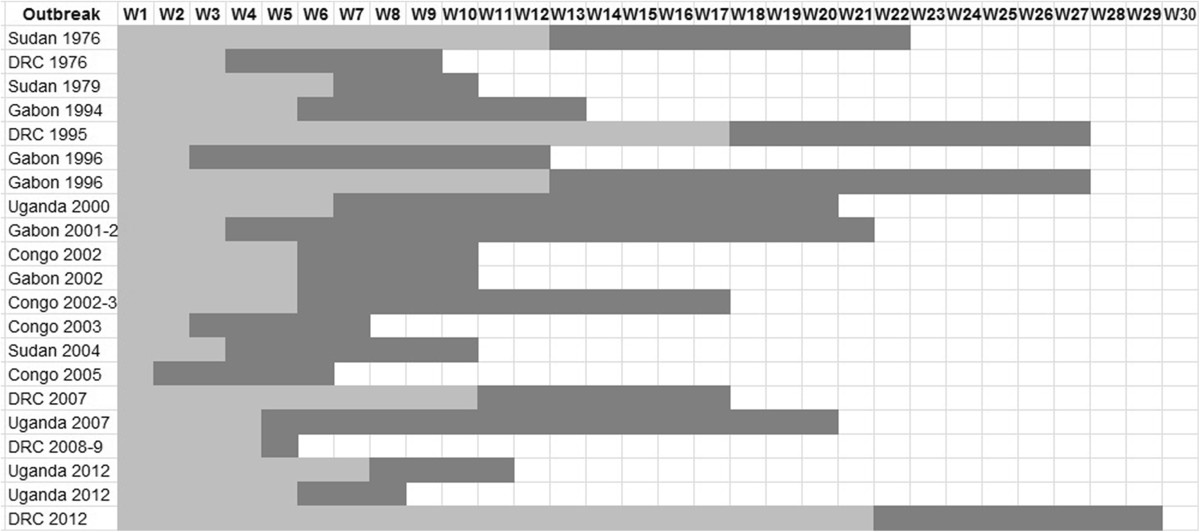
**Alert delay and duration of the African Ebola outbreaks.**

Beyond the diversity of African outbreaks, particularly regarding the incidence and duration of the epidemics (Table 5), we can notice that most of them presented only one source of infection accounting for the spread of the virus. As a result, it can be assumed that either the opportunities for human contact with the virus are rare, or the risk of contamination is limited. The period between the index case and the alert averages about two months. In addition, an equivalent period occurs between the alert and the last confirmed case (Figure 6).

**Table 5:** **Length of African Ebola outbreaks**

Outbreak	Index case	Alert	Last case	Length of outbreak*	Number of outbreak sources	References
Sudan 1976	Jun. 27, 1976	Sep. 15, 1976	Nov. 25, 1976	151 days	1	[25, 101]
DRC 1976	Sep. 1, 1976	Sep. 21, 1976	Nov. 5, 1976	66 days	1	[26, 102]
Sudan 1979	Jul. 31, 1979	Sep. 12, 1979	Oct. 6, 1979	67 days	1	[24, 103]
Gabon 1994-5	Nov. 13, 1994	Dec. 18, 1994^1^	Feb. 9, 1995	88 days	?	[67, 84, 104]
DRC 1995	Jan. 6, 1995	May 1, 1995	Jul. 16, 1995	191 days	1	[28, 87]
Gabon 1996	Jan. 31, 1996	Feb. 13, 1996	Mar. 12, 1996	83 days	1	[67, 88, 89]
Gabon 1996-7	Jul. 13, 1996	Oct. 5, 1996	Jan. 18, 1997	189 days	?	[67, 90, 91]
Uganda 2000-1	Aug. 30, 2000	Oct. 8, 2000	Jan. 14, 2001	137 days	1	[21, 105]
Gabon 2001-2	Oct. 25, 2001	Nov. 17, 2001	Mar. 22, 2002	148 days	5	[69, 34]
Congo 2001-2	ND	ND	ND	ND	?	[34]
Congo 2002	May 17, 2002	June 6, 2002	Jul. 25, 2002	69 days	1	[34]
Gabon 2002	May 17, 2002	Jun. 21, 2002	Jul. 25, 2002?	69 days?	1	[34]
Congo 2002-3	Dec. 25, 2002	Jan. 28, 2003	Apr. 22, 2003	118 days	3	[71]
Congo 2003	Oct. 11, 2003	Oct. 24, 2003	Dec. 2, 2003	52 days	1	[92]
Sudan 2004	Apr. 15, 2004	May 6, 2004	June 26, 2004	72 days	1	[93]
Congo 2005	Apr. 18, 2005	Apr. 26, 2005	May 27, 2005	39 days	1	[70]
DRC 2007	Jun. 12, 2007	Aug. 22, 2007	Oct. 10, 2007	120 days	1	[58, 19]
Uganda 2007-8	Aug. 20, 2007	Sep. 15, 2007	Jan. 8, 2008	141 days	1	[30, 33, 94]
DRC 2008-9	Nov. 27, 2008	Dec. 25, 2008	Jan. 1, 2009	34 days	1	[19]
Uganda 2012^2^	Jun. 11, 2012	Jul. 24, 2012	Aug. 24, 2012	74 days	1	[96, 97]
Uganda 2012^2^	Oct. 13, 2012	Nov. 14, 2012	Dec. 5, 2012	53 days	1	[97–99]
DRC 2012^2^	Mar. 20, 2012	Aug. 17, 2012	Oct. 11, 2012	206 days	?	[79, 100]

*Between the index case and recovery or death of the last case. ^1^First reported as yellow fever[85]. ^2^Partial unconsolidated data. ND: not determined.

The origin of the disease and its management are not perceived the same way by different people[77–79, 82]. At the beginning of epidemics, the disease is often confused with another endemic infection (such as malaria, dysentery, typhoid or yellow fever, influenza etc.), both by the general population and health personnel. When the incorrect causes are eliminated or diagnosis of EVD is confirmed, other explanations of cultural, religious, circumstantial or biomedical origins are proposed. It has been shown, in several epidemics, that affected people perceived the illness as divine punishment or evil spell, and even, sometimes, denied the disease itself[77–79]. Hesitations about diagnosis and the diversity of explanatory anthropological models result in the delay of the alert and difficulties in the implementation of measures against epidemics[79, 82, 92].

Finally, health authorities, for economic and political reasons, take late measures that only aim at minimizing the stress provoked by epidemics and avoiding panic. This results in inadequate or contradictory information, which adds to the general stress. Moreover, some measures taken to demonstrate the determination of the authorities are useless and counterproductive. The ban of gatherings and travel, border closures, police cordons and other initiatives only reinforce distrust of people *vis-à-vis* the authorities and health personnel.

### Management of outbreaks

The delay in clinical diagnosis and epidemic alert represent a major issue because it exacerbates the spread of the virus.

**Figure 7:**
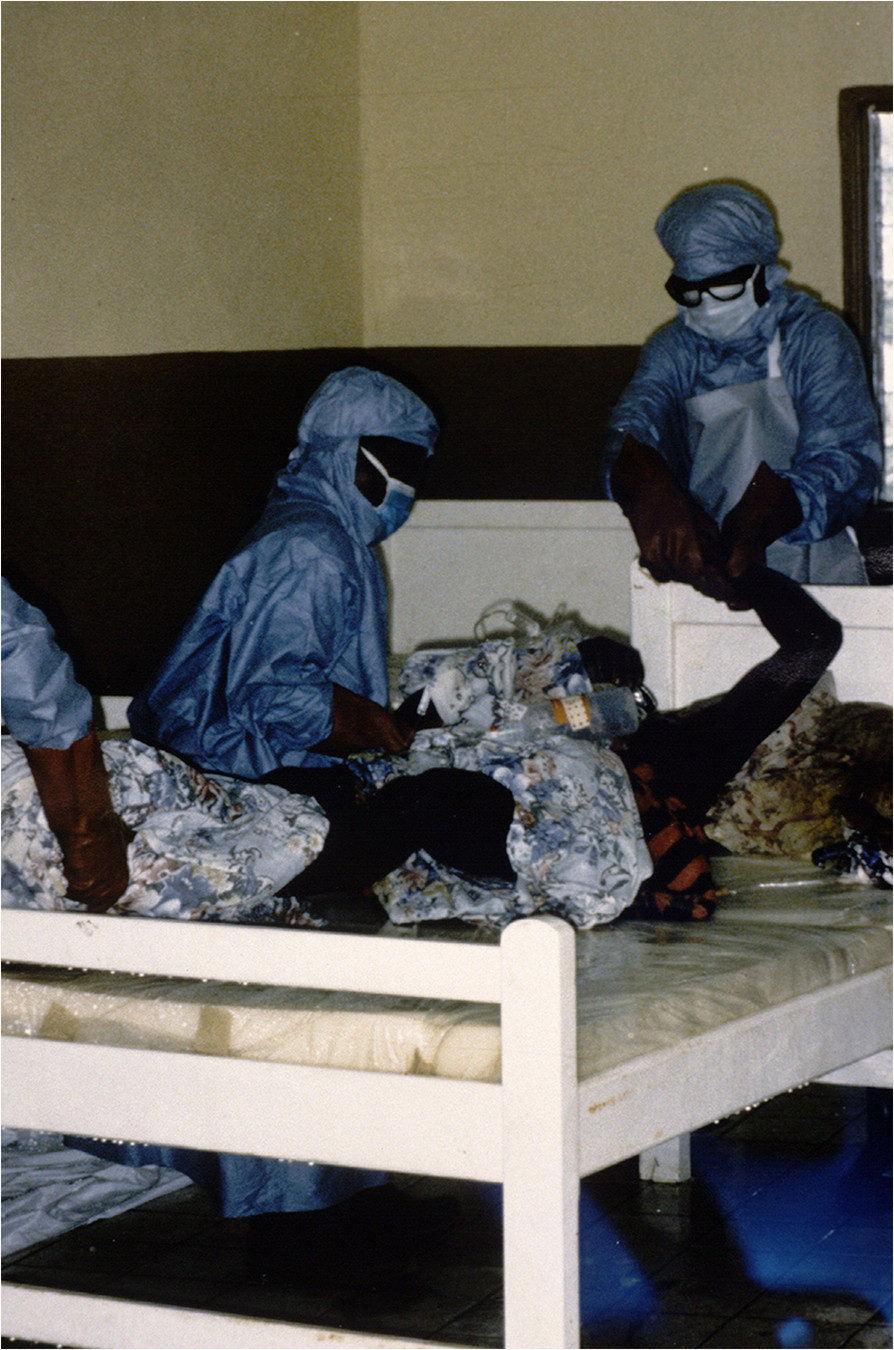
**Protective clothing worn by healthcare personnel during the care and handling of a patient (photo by Jean-Paul Gonzalez, ©IRD).**

**Figure 8:**
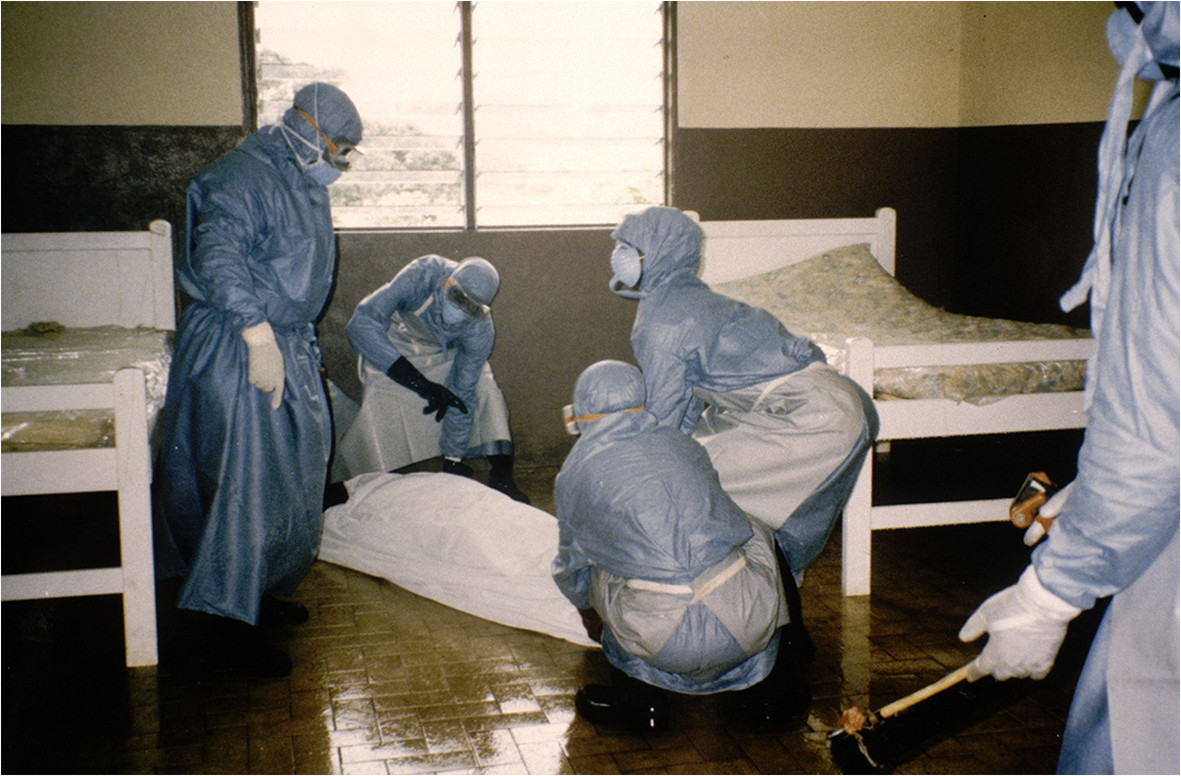
**Wrapping of the body in a mortuary sac (photo by Jean-Paul Gonzalez, ©IRD).**

**Figure 9:**
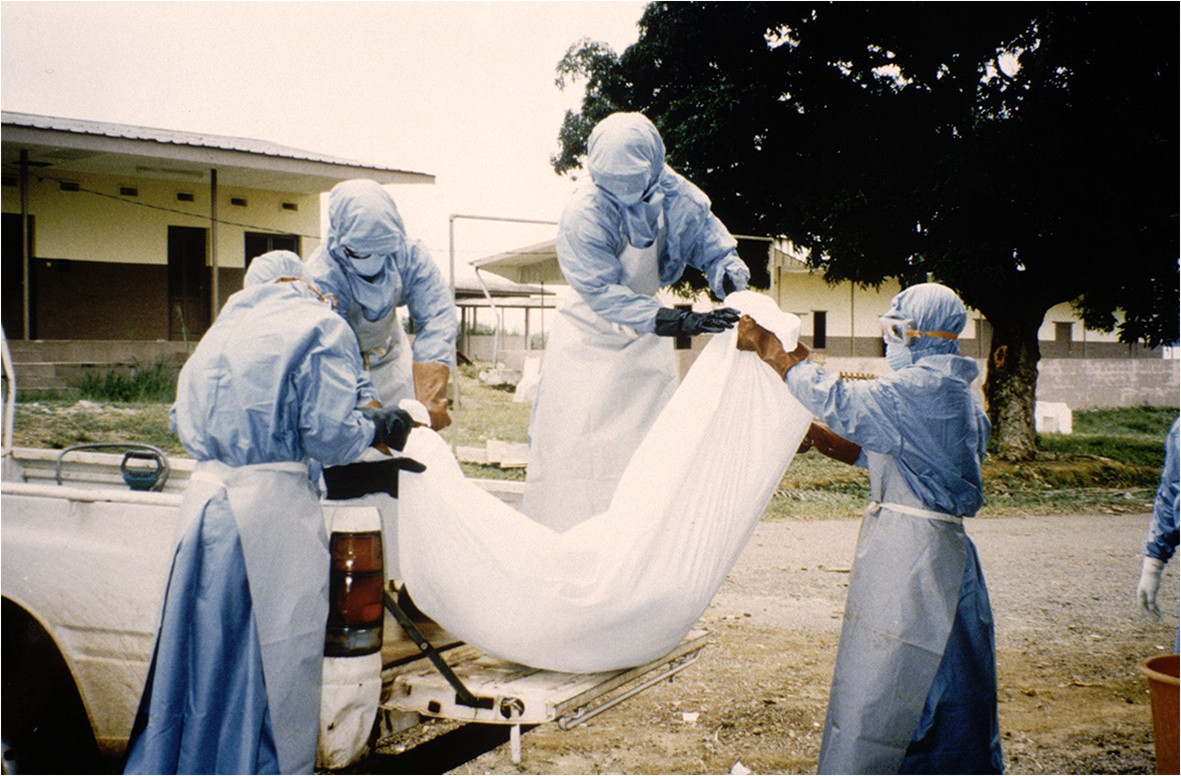
**Boarding of a corpse in the vehicle for transportation to the burial site (photo by Jean-Paul Gonzalez, ©IRD).**

In the absence of any vaccine or post-exposure treatments, it is essential to break the chain of transmission by acting directly on the causes and circumstances of outbreaks. All the precautions should be taken for patient care, management of dead bodies, burial and surveillance of contacts[106]. The Centers for Disease Control and the World Health Organization provide a manual in English, French and Portuguese (http://www.cdc.gov/vhf/abroad/vhf-manual.html) enabling health authorities to prepare for EVD outbreak management. After the description of the standard precautions regarding care of all patients to prevent infections (a brief summary of Good Medical Practice), the manual details the techniques used to control epidemics of EVD with many simple and practical hints:

- Clinical diagnosis of the disease.
- Isolation of the patient and planning the isolation area.Wearing appropriate clothing during treatment (Figure 7).

- Identification and monitoring of contacts.
- Disinfection and sterilization of equipment and facilities.
- Waste disposal.Preparation of the body (Figure 8).

- Transportation to the place of burial (Figure 9).

However, the management of cases and now well-known procedures face many difficulties that arise quickly in most outbreaks. In addition to the virulence of Ebola viruses and usual customs, it should be taken into account the changes in behavior induced by the fear generated by the epidemic and associated rumors.

Traditional practices regarding patient care and burial rituals often involve high risk conducts, such as washing and preparation of the body for exposure for several days, during which family and friends pay tribute by stroking or hugging the deceased[77–79, 82, 92]. Before appropriate measures are taken, which may take several weeks or months, the deceased persons are transported to their home community where people sometimes come from far away to attend the funeral and then go back home, which enables the spreading of the virus. The always frightening and often contradictory messages – and rumors – prompt patients to avoid going to the hospital due to fear of isolation and because of the lack of effective treatments. It becomes impossible to identify the cases, confirm diagnosis, protect and monitor contacts. Violent protests – with loss of life, involving sometimes the medical staff – have been reported in some outbreaks[71, 82, 92].

Other variables that hinder the adequate management of outbreaks include training of health workers who fail to immediately identify the disease, especially in areas where EVD has never been observed, either because of nonspecific clinical symptoms or lack of experience. Anyway, to date, there is no diagnostic test able to confirm an EVD suspicion. In addition, protective and disinfection equipment are usually absent and slow to become available in numerous health facilities[82]. Nosocomial transmission through contaminated and poorly (or not) sterilized medical instruments is a major multiplication factor in most outbreaks. Such picture is worsened by the fact that they hit in remote and poor regions.

Social mobilization is a key component because all stakeholders should be involved to enable pooling resources and optimizing management of epidemics[71]. The ethical aspects should not be overlooked. Isolation of patients, required to avoid contamination, should not be seen as segregation. The family should be able to see and talk to patients, even if they are prevented from touching them. Authorities and medical staff should comply with, as far as possible, funeral rites by providing body bags and coffins for the families[92]. For instance, decontamination will be presented as ablutions that can be associated with the current ritual; deceased’s clothes will be buried in the grave rather than burned to prevent stigmatization etc.[71, 77–79].

After the epidemic, it is important to reseal the bonds of community through social, cultural and sporting activities.

## Conclusion

Recently discovered in Central Africa, the Ebola viruses caused limited, but high-mortality epidemics. Fruit bats are probably the reservoir of the virus. The initial human infection results from contact with infected bush meat, and usually takes place in poor and inaccessible areas. Secondary transmission occurs during patient care at home, funeral rites or hospital dissemination due to the lack of preventive measures and because of inadequate training of health personnel with good medical practice.

The difficult diagnosis, the resistance of the population and the reluctance of authorities explain the slow and limited response to outbreaks, and the rapid spread of the epidemic that becomes hard to control.

In the absence of effective treatment, the discovery of an Ebola outbreak demands breaking the transmission chain at community and hospital levels, while respecting the traditions as much as possible, and the legitimate wish of relatives to accompany their sick or deceased relatives, in order to restore people confidence.

## Authors’ original submitted files for images

Below are the links to the authors’ original submitted files for images. Authors’ original file for figure 1 Authors’ original file for figure 2 Authors’ original file for figure 3 Authors’ original file for figure 4 Authors’ original file for figure 5 Authors’ original file for figure 6 Authors’ original file for figure 7 Authors’ original file for figure 8 Authors’ original file for figure 9
