# Supplementary figures and images for: Outbreaks of Ebola virus disease in Africa: the beginnings of a tragic saga
Source: J Venom Anim Toxins Incl Trop Dis. 2014 Oct 3;20:44. doi: 10.1186/1678-9199-20-44 (PMC4197285; doi:10.1186/1678-9199-20-44)

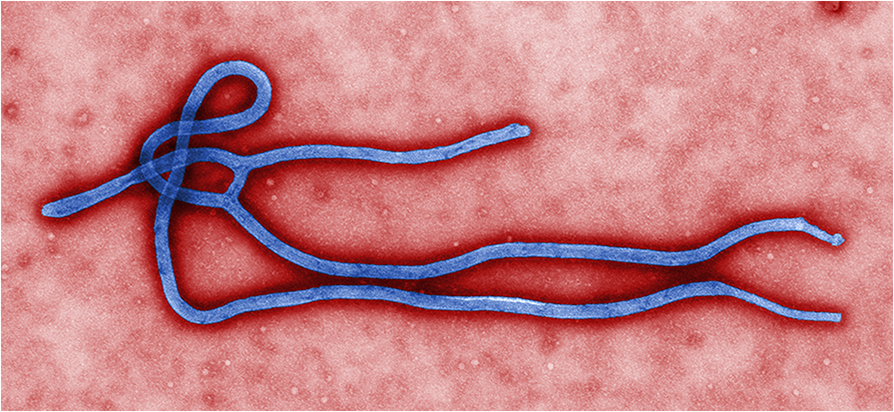

Supplement: Supplementary file 1 — Authors’ original file for figure 1 [file 40409_2014_70_MOESM1_ESM.tif]

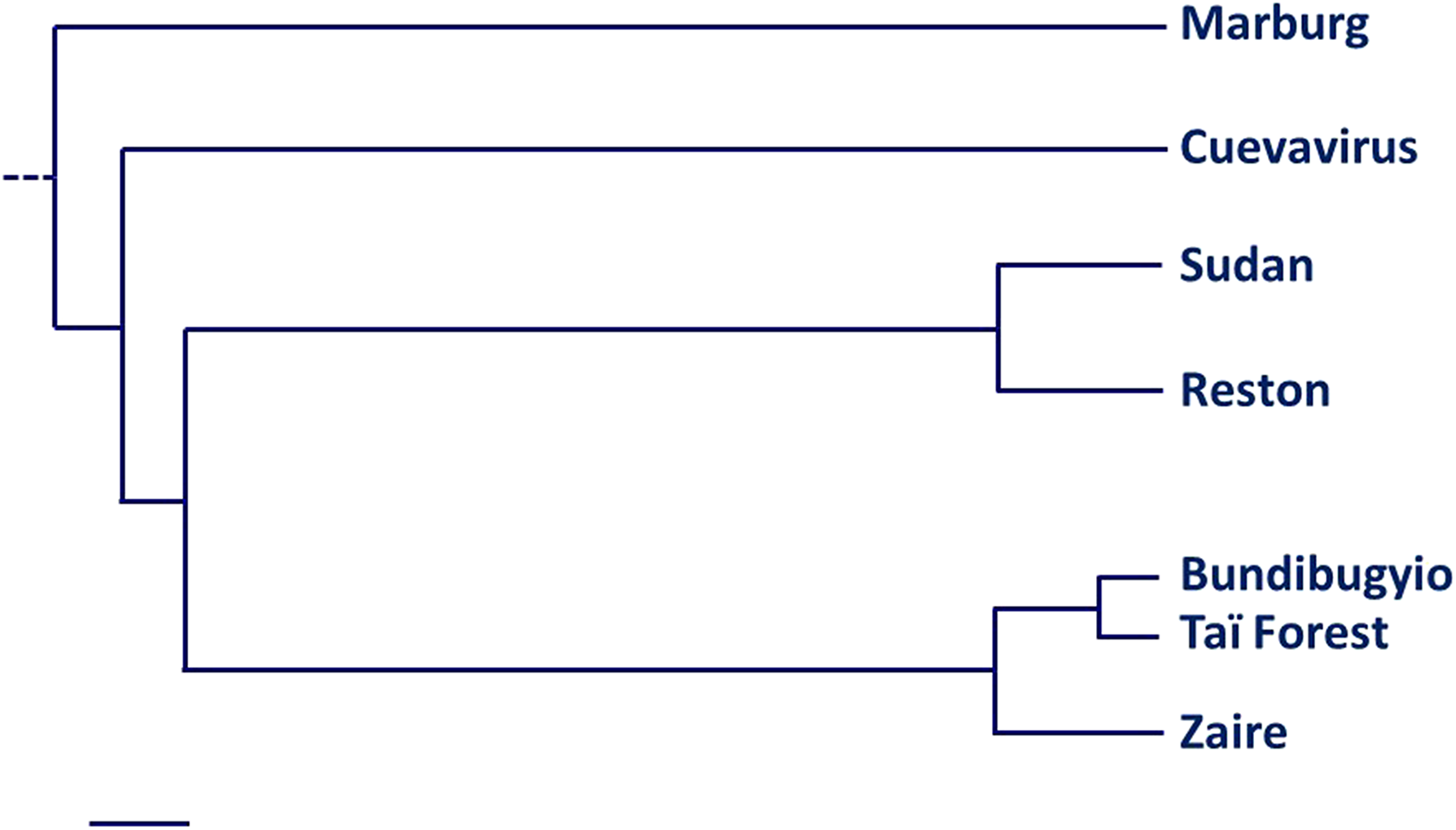

Supplement: Supplementary file 2 — Authors’ original file for figure 2 [file 40409_2014_70_MOESM2_ESM.tif]

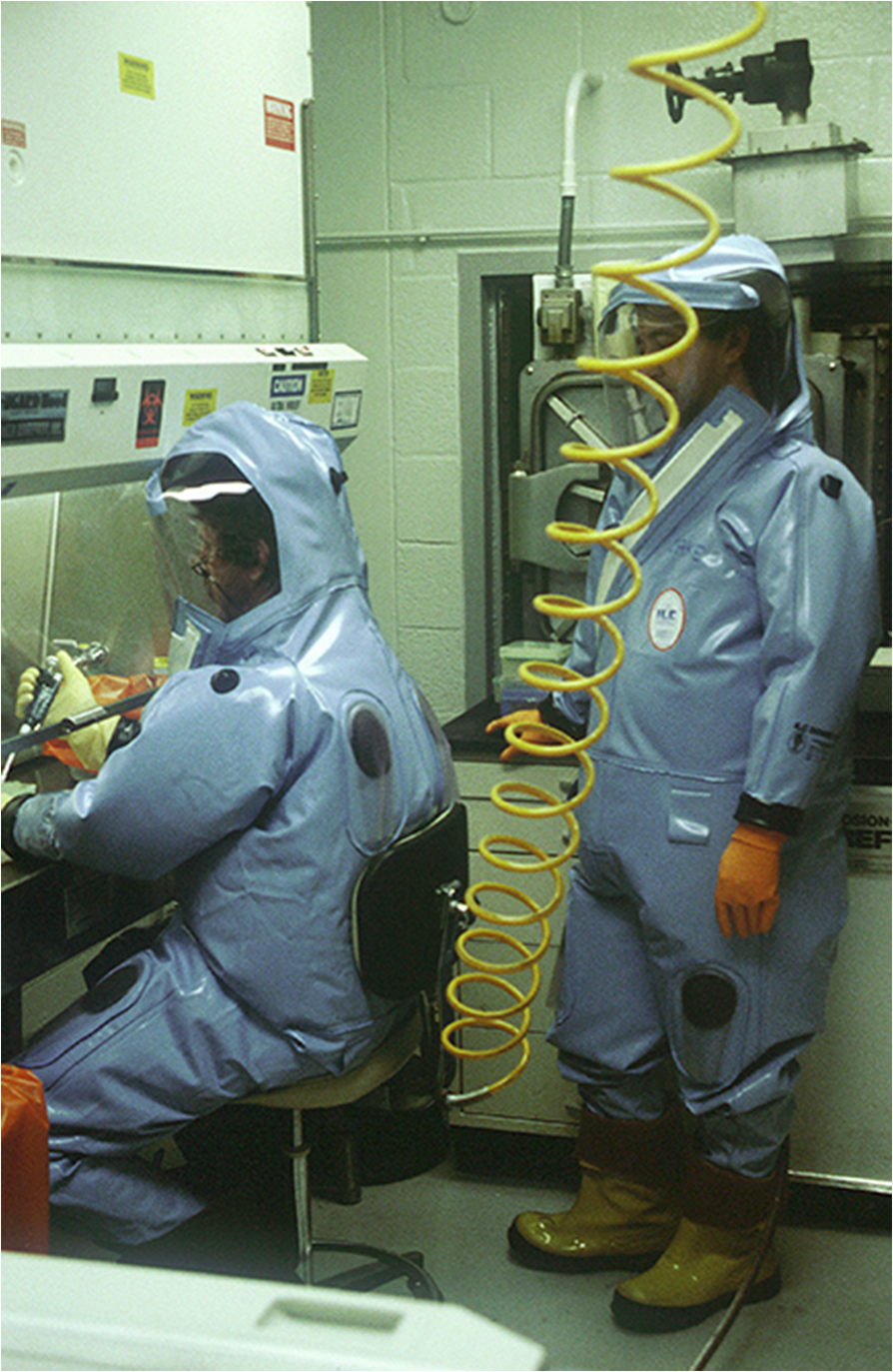

Supplement: Supplementary file 3 — Authors’ original file for figure 3 [file 40409_2014_70_MOESM3_ESM.tif]

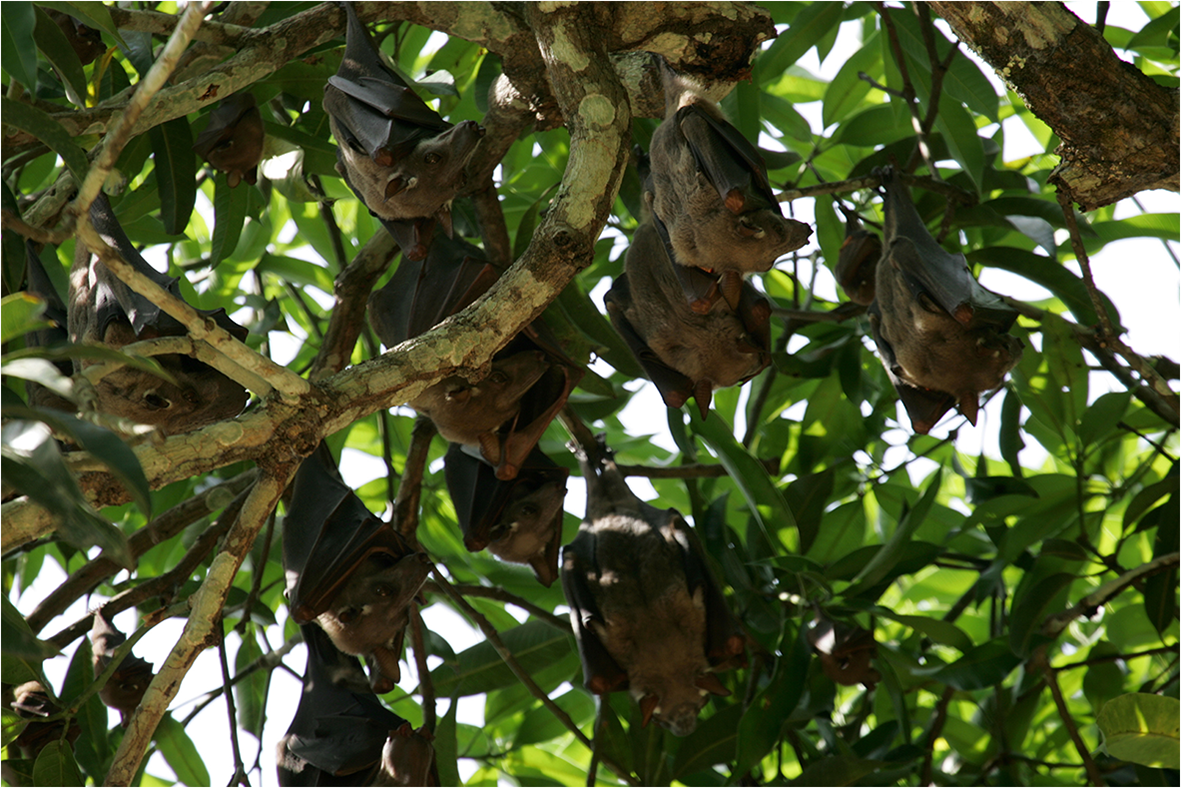

Supplement: Supplementary file 4 — Authors’ original file for figure 4 [file 40409_2014_70_MOESM4_ESM.tif]

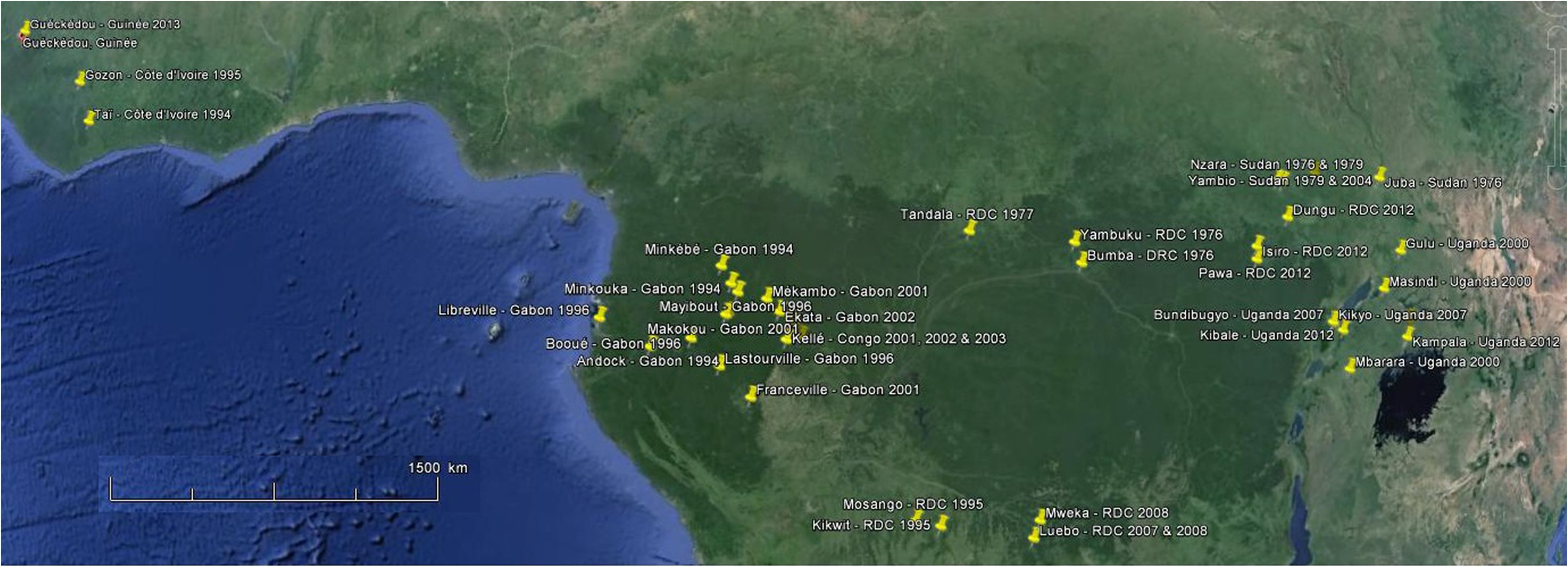

Supplement: Supplementary file 5 — Authors’ original file for figure 5 [file 40409_2014_70_MOESM5_ESM.tif]

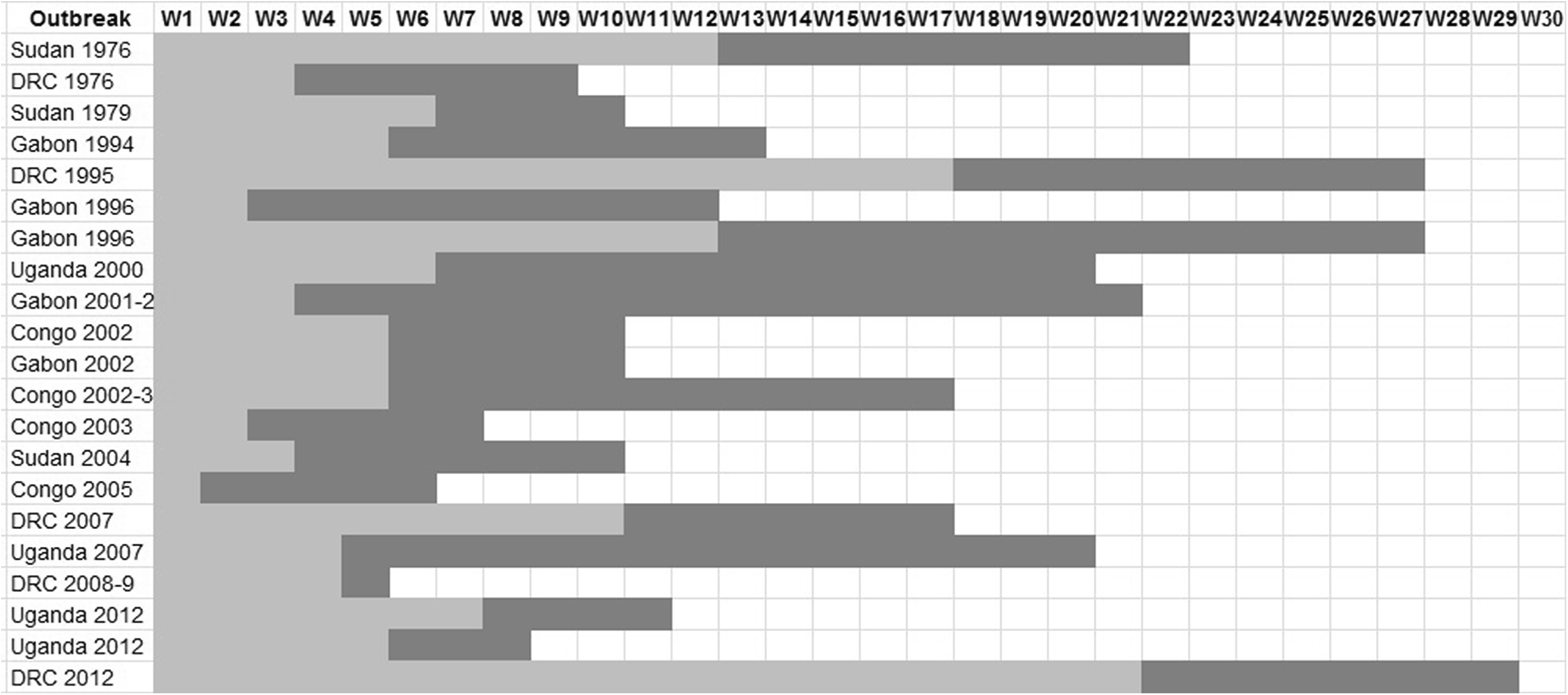

Supplement: Supplementary file 6 — Authors’ original file for figure 6 [file 40409_2014_70_MOESM6_ESM.tif]

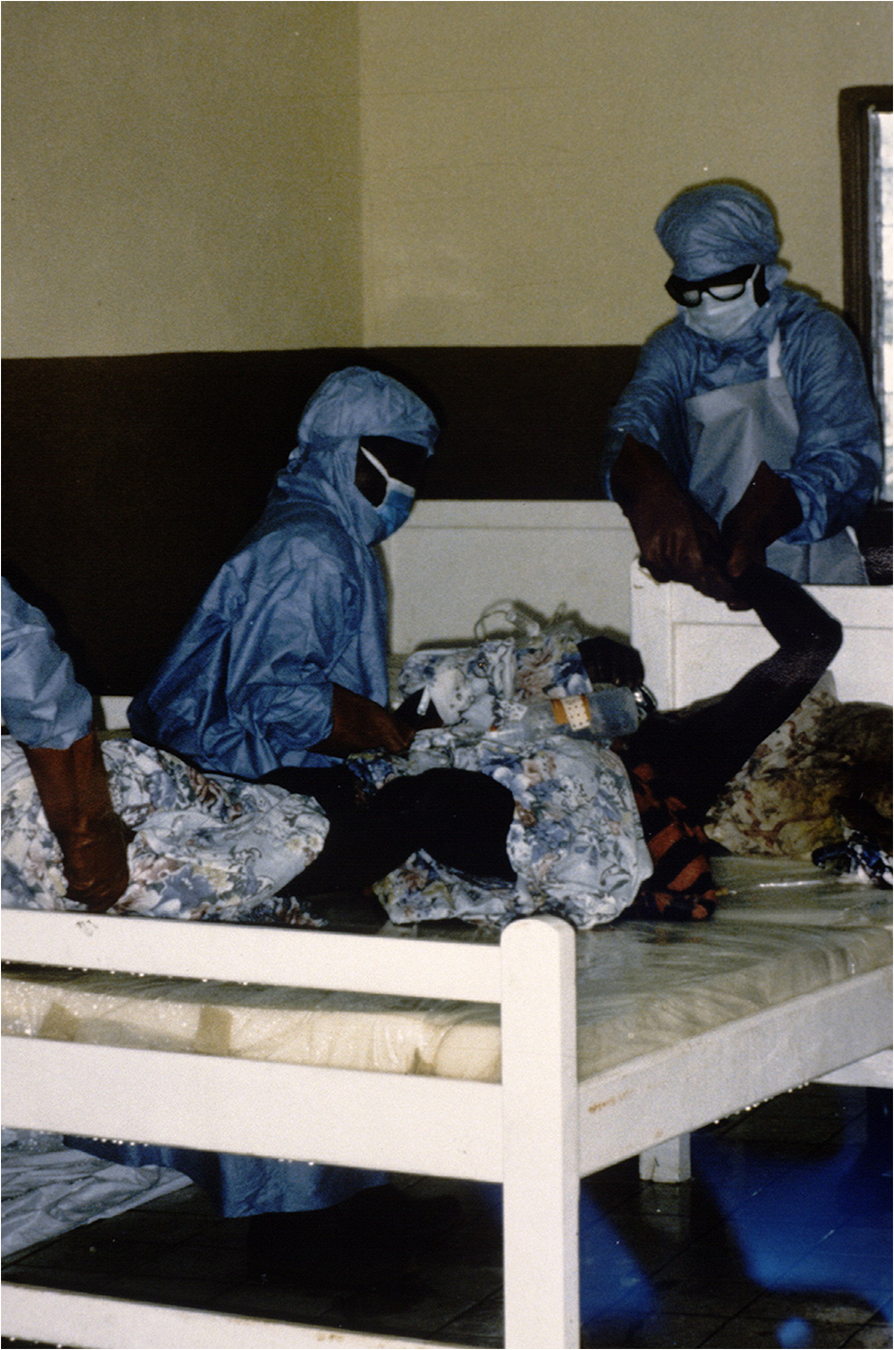

Supplement: Supplementary file 7 — Authors’ original file for figure 7 [file 40409_2014_70_MOESM7_ESM.tiff]

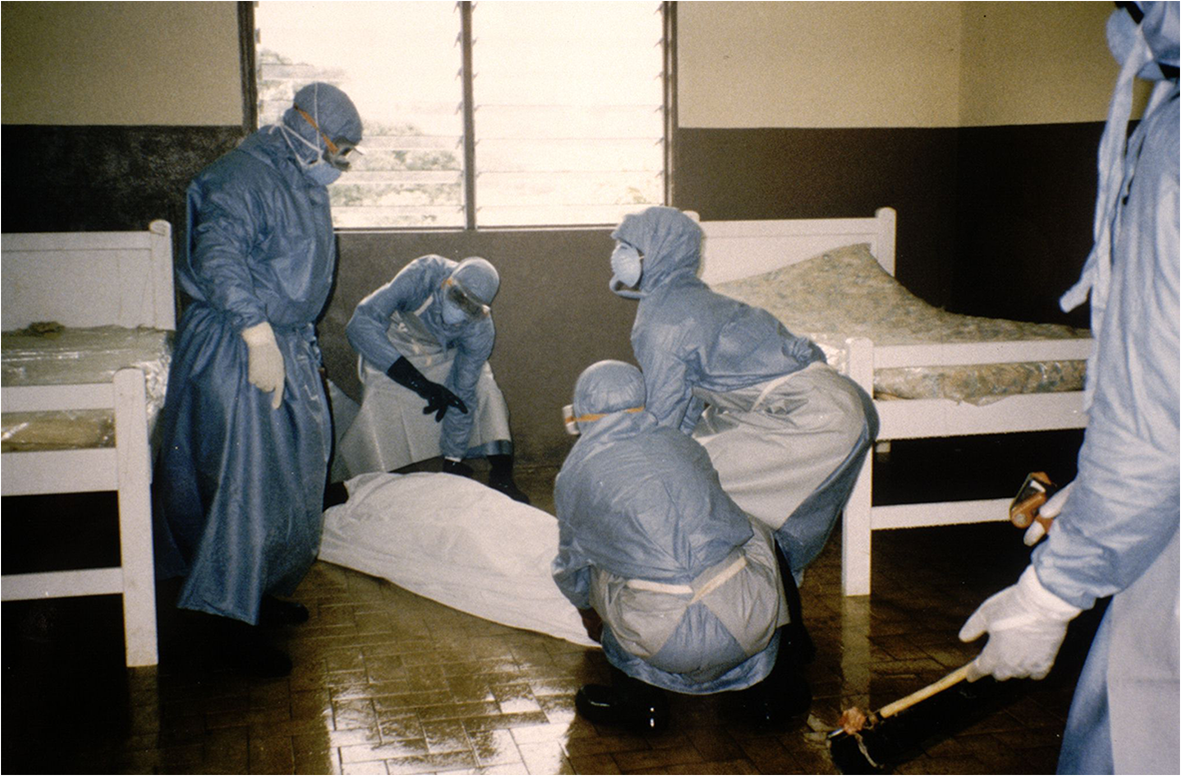

Supplement: Supplementary file 8 — Authors’ original file for figure 8 [file 40409_2014_70_MOESM8_ESM.tif]

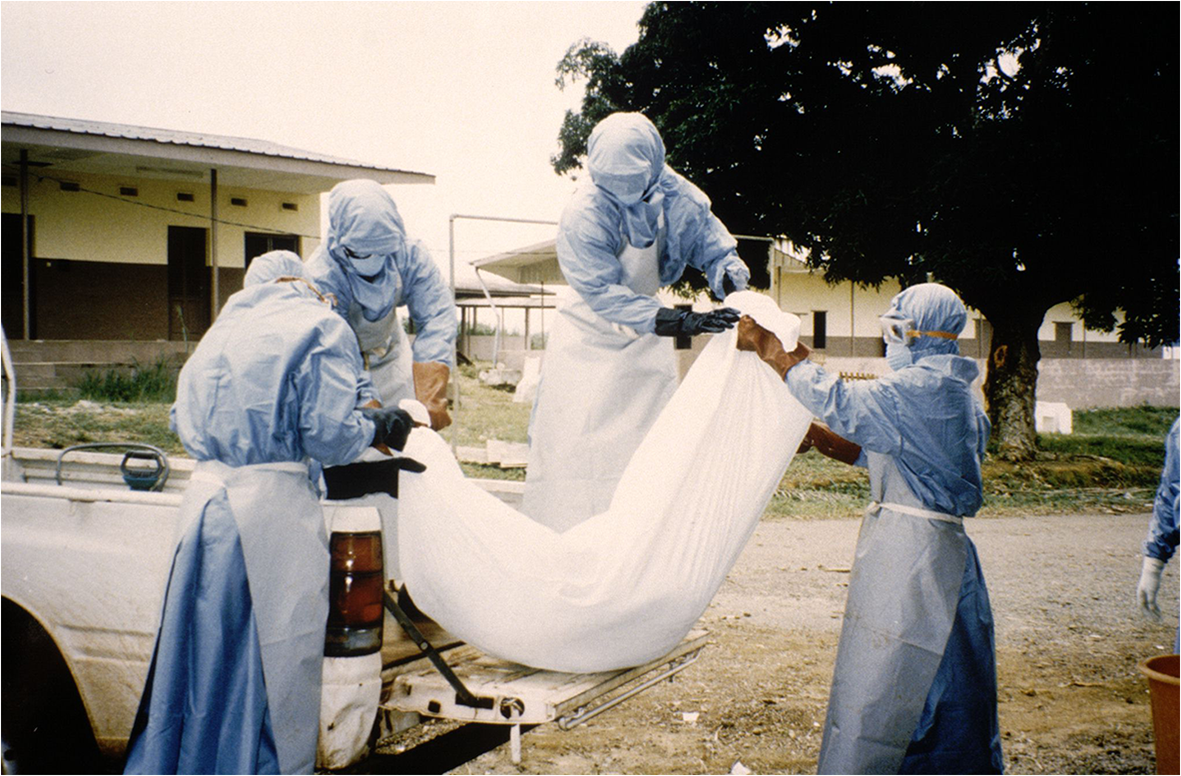

Supplement: Supplementary file 9 — Authors’ original file for figure 9 [file 40409_2014_70_MOESM9_ESM.tif]
